# Invasive or More Direct Measurements Can Provide an Objective Early-Stopping Ceiling for Training Deep Neural Networks on Non-invasive or Less-Direct Biomedical Data

**DOI:** 10.1007/s42979-022-01553-8

**Published:** 2023-01-12

**Authors:** Christopher W. Bartlett, Jamie Bossenbroek, Yukie Ueyama, Patricia McCallinhart, Olivia A. Peters, Donna A. Santillan, Mark K. Santillan, Aaron J. Trask, William C. Ray

**Affiliations:** 1 Department of Pediatrics, The Ohio State University College of Medicine, Columbus, OH USA; 2 The Steve and Cindy Rasmussen Institute for Genomic Medicine, Battelle Center for Computational Biology, The Abigail Wexner Research Institute at Nationwide Children’s Hospital, Columbus, OH USA; 3 Department of Computer Science and Engineering, The Ohio State University College of Engineering, Columbus, OH USA; 4 Center for Cardiovascular Research, The Abigail Wexner Research Institute at Nationwide Children’s Hospital, Columbus, OH USA; 5 Department of Obstetrics & Gynecology, University of Iowa Hospitals & Clinics, Iowa City, IA USA; 6 The Interdisciplinary Graduate Program in Biophysics, The Ohio State University Graduate School, Columbus, OH USA

**Keywords:** Machine learning, Health, Invasive, Non-invasive, Model, Overfitting

## Abstract

Early stopping is an extremely common tool to minimize overfitting, which would otherwise be a cause of poor generalization of the model to novel data. However, early stopping is a heuristic that, while effective, primarily relies on ad hoc parameters and metrics. Optimizing when to stop remains a challenge. In this paper, we suggest that for some biomedical applications, a natural dichotomy of invasive/non-invasive measurements, or more generally proximal vs distal measurements of a biological system can be exploited to provide objective advice on early stopping. We discuss the conditions where invasive measurements of a biological process should provide better predictions than non-invasive measurements, or at best offer parity. Hence, if data from an invasive measurement are available locally, or from the literature, that information can be leveraged to know with high certainty whether a model of non-invasive data is overfitted. We present paired invasive/non-invasive cardiac and coronary artery measurements from two mouse strains, one of which spontaneously develops type 2 diabetes, posed as a classification problem. Examination of the various stopping rules shows that generalization is reduced with more training epochs and commonly applied stopping rules give widely different generalization error estimates. The use of an empirically derived training ceiling is demonstrated to be helpful as added information to leverage early stopping in order to reduce overfitting.

## Introduction

Despite rapid advances in machine learning, solutions to the problem of overfitting remain primarily ad hoc. Caught between the horns of a dilemma, a data scientist usually wishes to maximize the predictive capability of a model, while avoiding over-learning the data and losing generality. This challenge may be faced without adequate information regarding both what is “good enough” for model performance, and what is “too good” and verging into the realm of overfitting. Across machine learning, poor generalization is dealt with by constraining the model fitting to favor simpler models, a process known as regularization. Some methods penalize the parameters directly while other methods penalize overfitting implicitly, such as randomly shutting down nodes while training a neural network, known as dropout.

Early stopping is another common regularization method. Early stopping is appealing because it does not make assumptions about the informational distribution of the model. It assumes only that the early model learns general features of the training data, and that it increasingly learns specific features of the data as additional training epochs are conducted. The simplest application is to train the network for many epochs, saving model weights at each epoch, and then to pick the epoch with the lowest validation error (and, therefore, the least generalization error). The goal of early stopping is to stop at the ideal epoch without the cost of generating the entire error validation curve. However, there currently does not appear to be a general solution for predicting ideal early-stopping points.

For some specific applications, such as medical imaging, we propose an empirical bound that can effectively be considered a hard ceiling on the best possible performance a deep neural network (DNN) could attain, in effect allowing us to know what is “too good” and, therefore, verging into the realm of overfitting. Such a ceiling offers guidance on when continued training is not advantageous, albeit under certain regularity conditions we will discuss below.

For biomedical problems, the availability of invasive measurements may provide insight into the information available in non-invasive measurements. We propose the following postulate about information content for machine learning as the premise of our contribution.

*A priori*, the information in a non-invasive, surface-measured correlate of some underlying biomechanical phenomenon cannot exceed the information content of an invasive measurement of the underlying phenomenon itself.

Not all variation is useful for prediction, and the predictive power of a system is limited by both the noise in the measurement system, the latent signal being measured, and any ambiguity or noise in the classification system for the desired output. We presume the following logic. To the extent that invasive measurements relate to the same or a highly correlated underlying phenomenon as a congruent non-invasive measurement, the invasive measurement should offer the better attainable predictive power. Therefore, when training a DNN on data from a non-invasive measure, we claim that going beyond the predictive ceiling bounded by the invasive measure’s performance is a clear indication of overtraining and poor generalization.

### Defining Quantitative Goals for Machine Learning

The concept of early stopping is often discussed in the DNN literature as a type of convergence criteria. When the loss in the validation dataset levels off across training epochs, the DNN has learned the generalizable aspects of the data. Continuing to train will only cause memorization effects, where aspects of the training data become more emphasized to the detriment of generalization.

In practice, the situation is more complex. Validation loss curves by epoch are not guaranteed to be smooth, and often are not. One might stop at a local minimum. Any convergence criteria formed through a simple heuristic may underperform. To train for more epochs offers the chance to see if the loss function has a lower local (or hopefully the global) minimum but can be costly and time-consuming. In addition, to be fully certain that the validation loss curve is accurate requires independent test data that has not been seen by the DNN classifier in training. Certainly, in biomedical applications, such hold-out data can be limited and potentially costly, such as when studying rare/uncommon disease populations.

The balance of finding empirical guidance regarding when to stop training a DNN versus how much test data is available is not quantitatively defined in the literature and remains an unsolved problem.

Early stopping is the best-known heuristic and many important attempts to formalize the concept have been put forward. For example, Prechelt defines a family of metrics [2], each of which could be used in an early-stopping rule. Both dataset sizes and computational power have grown exponentially since then, so the empirical evaluation of the best metric may be different today. In addition, several attempts to formalize both metrics and early-stopping algorithms that may perform well in our setting, have appeared in the literature for other specific applications [2, 3].

In this study, we offer a different point of view of the early stopping problem, borne from the authors’ experience with experimental systems: Invasive measurements in a biological system could offer the best attainable measures of the system’s intrinsics while non-invasive measurements are more distal, and can at best equal the predictive power of DNN’s trained on invasive measurements.

We present this as a form of outside knowledge to inform our early stopping rules. Having classifiers trained on invasive measurements as a quantitative benchmark provides an empirical ceiling for training non-invasive measurements. This assumes that invasive measurements of reasonable quality are, or have been, available for machine learning with appropriately vetted model performance. In the life sciences, this assumption is true with reasonable frequency. Over time, there have been many invasive studies of discrete biomechanical organismal systems. Sometimes these were performed to acquire primary or secondary data for a research study, some collected for standard-of-care record-keeping, and others acquired out of pure curiosity about biological function. Much of these data would be difficult to acquire prospectively, due to bioethics concerns, a limited understanding of the full utility of the invasively-acquired data, or the simple challenge of enrolling sufficient test subjects in a timely fashion. However, the wealth of outside knowledge that can be gleaned from such studies can significantly augment what can be learned from modern studies using less invasive techniques.

In what follows, we analyze both invasive and non-invasive measurements on the same animals in order to predict disease status. However in most research contexts, data from invasive measurement machine learning could be taken from the literature or developed from publicly available datasets.

### Related Work

The concept of early-stopping predates the current DNN literature and early attempts to define useful metrics for evaluating potential stopping points were defined prior to the recent rapid growth of available data (e.g., Prechelt’s work in 1998 [2]). Interestingly, the general ideas behind those metrics are still part of common practice today and are available in widely used packages for machine learning such as Tensorfow [4]. Early stopping uses training and validation datasets to assess changes in model generalization. When the validation error goes up, productive training is stopped.

Critically, the approach underlying all current methods, relies on analyzing the trajectory of the training results with the subject data itself. Different methods make different heuristic choices regarding what properties of the trajectory indicate that validation error has leveled off or begun increasing, but all base their decisions on the behavior of training on data of the same type and source as that to be learned.

For example, in many approaches, the number of epochs of stalled progress or increasing error during which training continues before early stopping is controlled by a parameter called “patience”. The patience metric approach is not computationally demanding, which is a strength of the approach [4, 5]. Using the patience approach, the DNN is trained, and for any epoch where the validation error is smaller than any previously observed, those model parameters are saved [6]. Once the generalization gap—the gap between the training error and validation error—increases to the point that further training seems unfruitful to continue, then the model parameters associated with the lowest validation error are chosen as the final classifier. Typical values for patience range from 3 to 6 epochs.

Many variations on the basic theme of early stopping continue to be developed. Much of the literature offers heuristics that are elucidated in a context-specific way. In breast cancer research, a rising trend in validation loss has been described but not quantitatively defined [7]. Overfitting in the context of feature selection had an early-stopping algorithm defined to reduce computing time per cross validation step [8]. In the context of fuzzy clustering coupled to a neural network, a patience value of 6 was recommended [9]. In fact, the patience value of 6 arises in other contexts too, including neural networks for computer vision [9, 10], a domain which is relevant for the present application. Metrics for early stopping have been derived that offer quantitative guidance. One example we adopt here comes from Deng and Kwok [3]. Their metric tunes what is considered an upward trend in the validation loss at each iteration.

Despite the broad variety of earlier work on early-stopping criteria, to date there has not been a systematic evaluation of early-stopping metrics. Each proposed solution is context dependent and represents an approximation of a consistent and reliable ceiling for early stopping. Table 1 summarizes the criteria applied to the training trajectory for each of the early stopping methods evaluated in this manuscript.

**Table 1:** Summary of the early-stopping methods used for comparison in this manuscript

Method	Stopping Criteria Heuristic
Generalization loss	Current loss divided by previous minimum loss exceeds threshold
Progress quotient_n_	GL smoothed over n previous epochs exceeds threshold
Patience_n_	*n* epochs of stalled progress or increasing error
Deng & Kwok	Increases patience when $\text {current loss} < 0.996 \times \text {previous minimum}$

In contrast to these approaches, in this manuscript we propose that in some cases there are sources of data external to the data to be learned, that can provide an objective ceiling for performance on the subject data, and, therefore, can provide an early-stopping criteria that does not depend on the subject-data training trajectory.

An abbreviated version of this work has been previously published in the Proceedings of the International Conference on Signal Processing and Multimedia Applications [1]. Here, we have extended our previous work by including analysis of a complementary dataset that provides additional evidence for the utility of determining objective informational ceilings for machine learning in other informational contexts. This extension further enables us to generalize the concept of invasive versus non-invasive measures, to the concept of closer to or more directly measuring, versus more distant from or more indirectly measuring, the underlying pathophysiology.

The remainder of this manuscript is organized as follows: “The Demonstration Problems” describes the problems we will use to demonstrate overtraining phenomena and the results of different early-stopping choices; “Data Sources” describes the data sources used in this study; “Analysis Framework” describes our analysis framework. “Experimental” describes the actual data extraction, DNN training and experimental results; our “Discussion” discusses our results and their import for early-stopping choices in DNN training.

## The Demonstration Problems

For our demonstration, we focus on Doppler (ECHO) sonography to measure blood flow. Doppler sonography measures the relative speed of movement of a target (in this case blood) compared to a reference probe. In the case of blood flow in living organisms, the probe is placed on the skin surface and it measures the difference in frequency between an emitted ultrasonic waveform and the return wave reflected from inside the body. Adjustment of the instrument enables focusing the region of interest at specific depths and on specific anatomy. Movement within that region of interest causes a Doppler shift in the reflected waveform’s frequency. By this approach, Doppler sonography provides a non-invasive inferred measurement of internal blood velocity in the direction towards or away from the probe tip.

We apply Doppler sonography to two demonstration datasets: changes in blood flow in the coronary microvasculature that are indicative of Coronary Microvascular Disease (CMD), and changes in blood flow in the umbilical artery (UA) that are indicative of Intrauterine Growth Restriction (IUGR).

These two demonstrations offer complimentary perspectives on the analysis of Doppler sonography data. First, we discuss non-invasive measurements of blood flow in the heart that will later be compared to invasive measurements from the heart to predict disease state. Second we continue this logic of “the closer to the pathophysiology the better” by comparing non-invasive blood flow measurements of Uterine Artery (UA) flow pathology, to even less-direct clinical data obtained from medical histories and simple office procedures (e.g., blood pressure). In the second case, neither the sonography nor the clinical data are obtained invasively, but the sonography is closer to the physiology, and, therefore, can be used to inform early stopping when applying machine learning to the clinical data.

### Echocardiography in Coronary Microvascular Disease

**Fig. 1:**
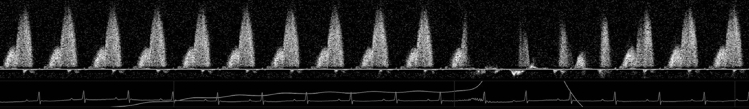
Transthoracic Doppler echocardiography (TTDE) data are acquired as a video, assembled into an image, each vertical slice of which is a greyscale histogram of the Doppler blood-flow velocities at that timepoint. Many sources of noise are layered onto the Doppler signal, so there is no internal reference to inform machine learning regarding the true information content. In this typical recording of 18 heartbeats, the data recorded for the first 10 beats represent physiologically realistic flow patterns, while the 11th through 16th beats display corrupted data due to movement of the transducer relative to the vessel being monitored. Electrocardiogram and respiratory recordings underlie the TTDE signal and assist in indexing the heart beat and identifying when predictable physiological phenomena such as breathing have occluded the TTDE data. Image from Bartlett et al. [1]

Coronary microvascular disease (CMD) is notoriously difficult to diagnose with non-invasive approaches. Current methods utilize only the peak velocity of the coronary flow pattern, and have poor predictive power [11]. TTDE data are typically acquired as a video of the time-varying Doppler signal, and a summary image from a typical TTDE experiment (video fused into a single image in a fashion analogous to a moving-slit aperture) is shown in Fig. 1. There are currently no non-invasive methods that incorporate the coronary flow pattern over a complete cardiac cycle to definitively assess and predict the development of CMD.

Coronary blood flow (CBF) reflects the summation of flow in the coronary microcirculation, and we have begun to harness the uniqueness of the CBF pattern under varying flow and disease conditions (e.g., type 2 diabetes) to determine whether it might harbor novel clues leading to the early detection of CMD. Previous studies indicate an early onset of CMD in both type 2 diabetes mellitus (T2DM) and metabolic syndrome (MetS) that occurs prior to the onset of macrovascular complications (16 weeks in T2DM db/db mice). This results in blood flow impairments and alterations in coronary resistance microvessel (CRM) structure, function, and biomechanics [12–21]. Collectively, these data strongly suggest an early onset of CMD, and, therefore, sub-clinical heart disease, in T2DM and MetS [15]. Importantly, Sunyecz et al. uncovered innovative correlations between CRM structure/biomechanics and newly-defined features of the coronary flow pattern [11], some of which were unique to normal or diabetic mice.

We have initially utilized the CBF features from [11], in the presence and absence of other factors such as cardiac function, to develop a mathematical model that defines 6 simple factors that contain predictive information on normal vs. diabetic coronary flow patterns. Utilizing a multidisciplinary approach, we sought to test whether the elements that influence coronary flow patterning would be useful in the direct assessment of CMD using computational modeling. We tested this utilizing non-invasive transthoracic Doppler echocardiography of coronary flow combined with simultaneous invasive cardiac pressure–volume loop (PV-loop) assessment of cardiac function.

**Fig. 2:**
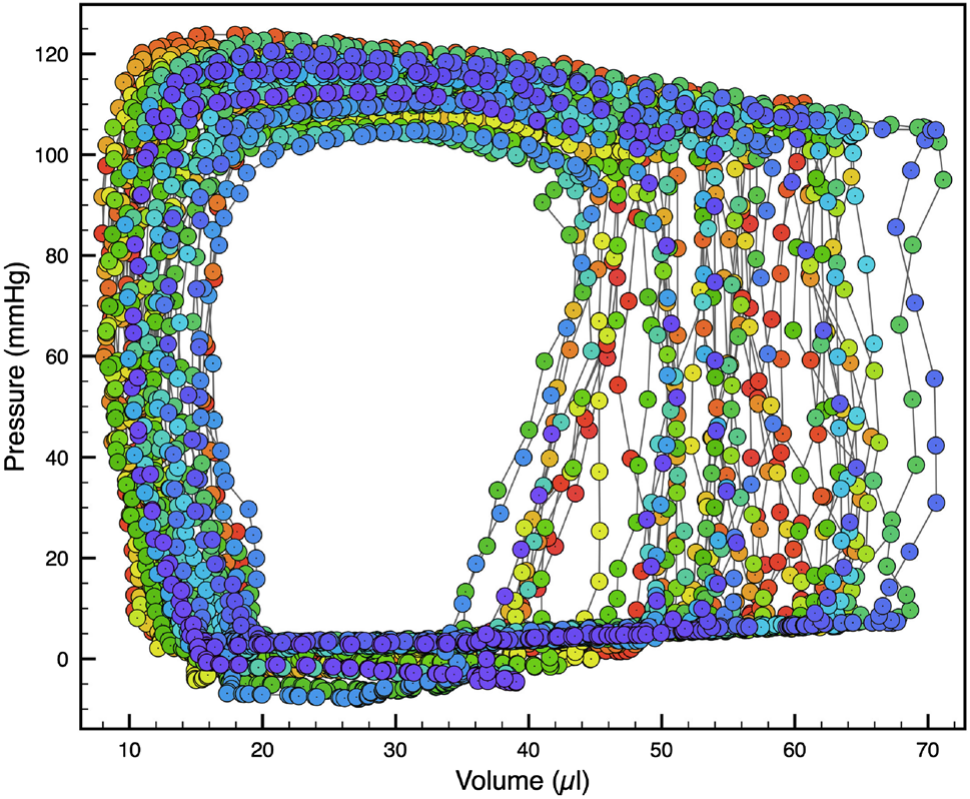
A typical pressure–volume “loop” (PV-loop) dataset. PV-loops are created by measuring paired values of pressure and volume in the left ventricle at 1000 Hz. The “loop” shape seen in PV-loop data can be understood in terms of the properties of a heart beat. Starting from the lower left, the low-pressure filling, followed by a near-fixed-volume increase in pressure, followed by a fixed pressure decrease in volume, and then a relaxation to baseline pressure to fill again, completes a single beat of the heart. Measured PV values over 46 heart beats are colored temporally in the figure on a rainbow gradient from Red (initial beat) to Indigo (last beat). PV-loops are not identical beat-to-beat due to real physiological differences in the beat-to-beat filling and contraction of the heart. Image from Bartlett et al. [1]

In contrast with TTDE data which are acquired as a video using an externally applied transducer, pressure–volume loop data are acquired as paired pressure–volume measurements using a probe inserted invasively into the heart. PV-loop data provide a completely different variety of data about cardiac function and the state of the cardiac microvasculature, from that obtainable through TTDE. A typical PV-loop recording is shown in Fig. 2.

### Umbilical Artery Doppler Flow in Intrauterine Growth Restriction

Intrauterine Growth Restriction (IUGR) is diagnosed in 23.8% of fetuses and it has clear ramifications. It leads to significant perinatal morbidity and mortality, birth hypoxia, impaired neurodevelopment, and metabolic syndrome in adult life [22, 23]. Standard practice is that non-invasive umbilical artery Doppler flow (UADF) measurement is the only Doppler measurement that should be used for IUGR monitoring [24]. UADF metrics showing absent or reversed blood flow during the diastolic phase of the cardiac cycle are associated with stillbirth. However, UADF metrics that would usually be considered to be worsening—such as elevations in the ratio of systolic blood flow (i.e., blood flow when the heart contracts) to diastolic blood flow (flow when the heart relaxes)—are not clearly predictive of poor outcomes [25]. As a result, clinical factors such as pregnancy-associated high blood pressure (pre-eclampsia), ultrasound measurements of fetal size and blood pressure are the current gold standard for prediction of IUGR. The use of simple UADF metrics only slightly improves the prediction of poor neonatal outcomes over clinical data alone (AUC increase from 0.74 to 0.82) [26]. The case for machine learning to improve the predictions of IUGR are self-evident. Machine learning can extract predictive information from sonographic image data, and learn how to predict which pregnancies can progress to IUGR.

## Data Sources

### Coronary Microvascular Disease

Two strains of mice that were 16 weeks old were housed under a 12-h light/dark cycle at $22^{\circ }$ C and 60% humidity. The two strains were normal control mice ($n=35$) and type 2 diabetic (DB) mice ($n=42$) (Jackson Laboratories). Mice were fed standard laboratory mice chow and allowed access to water *ad libitum.* This study was conducted in accordance with the NIH Guidelines and was approved by the Institutional Animal Care and Use Committee at the Abigail Wexner Research Institute at Nationwide Children’s Hospital.

#### TTDE Data (Non-invasive)

Transthoracic Doppler echocardiography (TTDE) video files of left main coronary blood flow with $\approx 20$ distinct cardiac cycles each were acquired from both groups of mice at baseline (1% isoflurane anesthesia) and hyperemic (increased blood flow measured at 3% isoflurane anesthesia) conditions following the protocol described by the Trask lab [11, 14, 27]. These videos were exported as.avi files from the Vevo2100 software and analyzed using an in-house Python script for data pre-processing. A summary image from a typical TTDE experiment is shown in Fig. 1.

#### PV-Loop Data (Invasive)

Invasive hemodynamic measures of cardiac function were terminally performed immediately following echocardiographic analysis as described by Trask et al. [28]. During the terminal experiment, mice continued to be anesthetized with isoflurane (2%) in 100% oxygen followed by tracheotomy and ventilated with a positive-pressure ventilator (Model SAR-830P, CWE, Inc.). A 1.2F combined conductance catheter-micromanometer (Models FTH-1212B-3518 and FTH-1212B-4018, Transonic SciSense, London, ON, Canada) connected to a pressure-conductance unit (Transonic SciSense, London, ON, Canada) and data acquisition system (PowerLab, AD Instruments, Colorado Springs, CO) was inserted into the right carotid artery and advanced past the aortic valve into the left ventricle. Pressure–volume loops were recorded off the ventilator for $\le 10$ seconds at baseline and during reduced preload by gently occluding the inferior vena cava with a cotton swab. We used approximately 30 measures obtained from invasive PV-loop measurements for our study. A typical PV-loop recording is shown in Fig. 2.

#### Post-processed Data

Each TTDE image contained a varying number of heartbeats (with an average of 22.63 ${\displaystyle \pm }$7.13 heartbeats per image) with low noise that were suitable for analysis. The number of heartbeats for analysis per group was 2810 for control and 3021 for DB. TTDE data were pre-processed as described by Sunyecz et al. [11].

### Intrauterine Growth Restriction

We obtained clinical, diagnostic, imaging and physiological data from 209 obstetrical patients receiving pregnancy care at the University of Iowa Hospitals and Clinics as part of an ongoing pilot study of IUGR. Images and clinical data were obtained through the Maternal Child Knowledgebase (MCK). The MCK is a transformative dataset which integrates maternal and child data from every pregnancy that received care at the University of Iowa Hospitals and Clinics since 2010. The MCK was queried for records that meet these inclusion/exclusion criteria: (1) the record must include (a) UADF measurements, (b) complete maternal-child data with regards to outcomes (including but not limited to diagnoses, vital sign information, medications, and procedures for the maternal-fetal dyad), (c) meta-data on the ultrasonograms to include sonographer, provider, machine make and model, time/date image was taken and (2) sonogram images must have native binary image meta-data. Using ICD-10 codes (O36*, P05*, Z03*, Z36*), a total of 138 cases with the diagnosis of IUGR were identified and validated by co-author MKS who is a board-certified maternal-fetal medicine subspecialist. A total of 71 controls were matched to the cases and then clinically validated (also by MKS).

#### Clinical Data (Non-invasive)

The ultrasound reports and the corresponding medical records of this cohort of patients were utilized to extract pertinent data including demographics, medical and obstetrical history data. The presence of IUGR ($Estimated Fetal Weight < 10\text {th} percentile$) was extracted as dichotomous variables ($0 = no IUGR$, $1 = UIGR$). Additional clinical variables included information about the pregnancy (e.g., gravida, parity, covariate diagnoses, medications, and blood pressure of the mother) and child characteristics (e.g., mode of delivery, APGAR scores, NICU admission, and sex).

#### UADF Data (Non-invasive but Directly Physiology-Based)

**Fig. 3:**
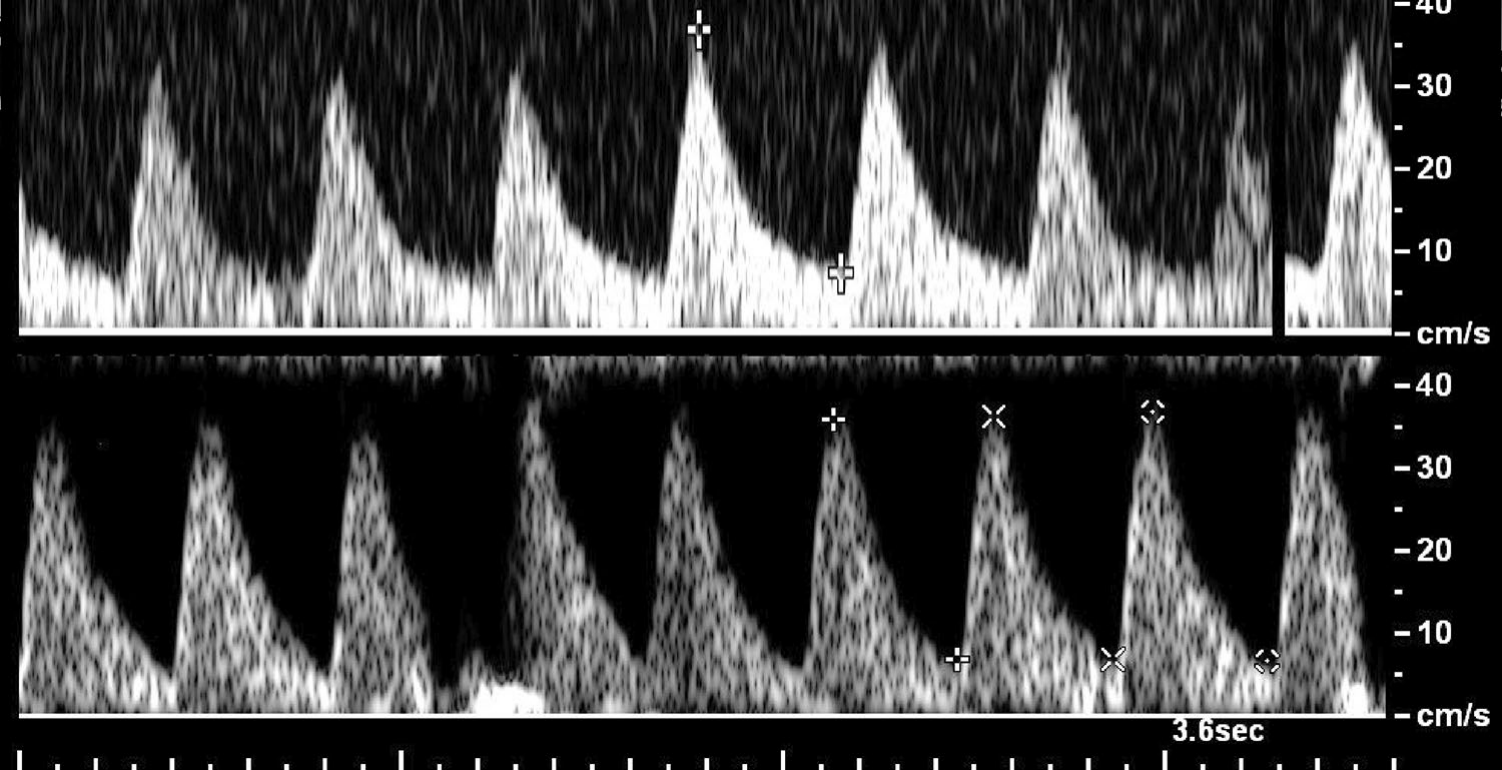
Umbilical artery Doppler flow sonograms from a normally developing pregnancy (top) and from a pregnancy developing intrauterine growth restriction (bottom). The average systolic to diastolic ratio—which is the current Doppler standard for predicting IUGR—in the top image is approximately 5.1, and in the lower image approximately 5.3 (essentially they are indistinguishable), yet preliminary data demonstrate that ML can differentiate between these and other similar UADF images with over 90% accuracy

Umbilical artery Doppler flow sonography (UADF) video files of the umbilical blood flow with three to seven distinct cardiac cycles each were acquired from patients in the clinic at the University of Iowa. Screenshots of heart cycles were annotated for clinical variables, de-identified, and the heart cycles were binned by systole and diastole for analysis. Figure 3 shows a comparison between the UADF Doppler sonograms of a normally developing pregnancy and one from a pregnancy with IUGR. These images look to a non-expert to be almost identical, and the only clinically accepted variable predicting IUGR that can be extracted from them (the systolic to diastolic velocity ratio) differs by far less than the individual-to-individual variation in either controls or cases. Despite this surface similarity, ML on UADF sonograms can distinguish between the normal and IUGR images with over 90% accuracy.

#### Post-processed Data

Each UADF image contained one heartbeat’s cycle with varying numbers of images per person (with an average of 6.5 heartbeats per person) with low noise that were suitable for analysis. The number of heartbeats for analysis per group was 215 for typical pregnancy and 741 for IUGR. IUGR data were pre-processed using an adaptation of our previous work [11].

## Analysis Framework

Our framework consists of deep learning to predict binary classes in two biomedical problems: coronary microvascular disease, and interuterine growth restriction.

Invasive measures can be more informative than non-invasive measures and, therefore, this is a readily identifiable distinction for analysts. However, here we introduce an abstracted dichotomy, that of more-direct/more informative, versus less-direct/less informative data sources. In this way, we offer an abstraction one step removed from the invasive/non-invasive dichotomy.

We first train using a more direct, more informative data source, and use the training performance of that model as an objective ceiling for early stopping when training on a less direct, less informative data source with the same goal. In biomedical research, direct measures of physiology can be (but are not uniformly) more informative than indirect clinical measures from patient history and physical examinations. We have chosen demonstration problems that show both these types of distinctions.

In both demonstration cases, we first trained on measures are closer to the underlying pathophysiology than the second dataset on which we wish to train. It is important to note that while this might appear to simply push the problem of determining a training ceiling onto a different ML training ceiling problem, we will show that the invasive PV-loop data are much more amenable to classification by simple regression. Therefore, training the DNN for the PV-loop data was compared to logistic regression to show that the DNN performance is approximately optimal given the highly informative nature of invasive measurements.

In many biological systems, the literature contains well-studied quantifications of the information content available for various invasive measures, and these may be used as ceilings for non-invasive work on those systems in lieu of performing an actual paired invasive study. Performance from training a DNN using the non-invasive data to classify control versus DB mice was compared to the invasive measurement performance ceiling to assess if overtraining has occurred. We go on to show that using both PV-loop and ECHO data in a DNN does not improve classification, indicating that no new additional information relevant to the classification is offered by the non-invasive measurement.

In addition, we tested several early-stopping metrics from the literature to assess their performance in this setting and to determine whether they can be misleading, relative to the empirical ceiling. In all analyses, data were split 80% training, 16% validation (used for testing generalization error each epoch), and 4% for the final out-of-sample test dataset. No outlier removal was applied as the exploratory analysis did not indicate any clear cases of outliers. The data were approximately balanced (see above), which is consistent with our experimental animal design. Our DNN implementation was in TensorFlow [4] and logistic regression was performed in scikit-learn [29].

### Analysis of Coronary Microvascular Data

For the coronary microvascular disease experiment, each mouse had both a non-invasive cardiac ECHO and paired invasive catheterization that obtained left ventricular pressure–volume (PV) loops. The ECHO data are non-invasive Doppler-sonographic measurements of coronary blood flow, while the PV-loops are direct invasive measurements of the pressure and volume in the heart. The volumetric change of the heart, and the pressure produced ultimately influence the coronary blood flow, so the flow being measured by the non-invasive ECHO method is highly correlated to these invasive measures. The two conditions for the DNN to classify are normal control versus DB mouse strains.

Diabetes changes cardiovascular structure, function, and stiffness, directly influencing the cardiac pressure–volume relationship and coronary blood flow. For both ECHO and PV-loop data, every heartbeat provides an iteration of cardiac data. The images from each mouse ECHO contain many heartbeats where each provides information for training the DNN. Labels for classification derive from the type of mouse.

### Analysis of Umbilical Artery Data

An evaluation for a single umbilical artery was performed during each routine anatomy scan. The umbilical artery Doppler flow heartbeats were extracted from screen captures of the sonography software. As with coronary flow above, each heartbeat provides an iteration of Doppler flow data that can be used for classification. Labels for classification derive from the diagnosis of the patient (typical pregnancy or IUGR).

## Experimental

### Establishing a Ceiling Using Invasive Data

Invasive PV-loop data were used to classify mouse strain in a retrospective diagnostic study design. Heartbeats were randomly sampled across mouse strain for each batch. No data augmentation was applied. Batch size was set to 32 and the learning rate was 0.01 as part of the Adam algorithm [30]. The loss function was binary cross-entropy on a DNN with six hidden layers. Training was conducted over 2000 epochs and the early-stopping procedure using a patience of 6 was applied post hoc. Waiting longer in the training than epoch 117 would not improve predictions and final test accuracy was 0.972. Logistic regression with recursive feature elimination (RFE) was performed on the PV-loop dataset. RFE selectively dropped four physiological parameters from the final model. Logistic regression of the RFE selected model gave similar prediction accuracy as the DNN ($accuracy = 0.971$). As expected, results of the logistic regression indicated a significant association of the PV-loop physiological parameters with mouse strain ($\chi ^2=7338.1$, $df=15$, $p<0.0001$). As the logistic regression model is less complicated than the DNN, this result highlights the high information content of the PV-loop data, making the less complicated regression model adequately powered to have similar predictive accuracy. From this, we infer that training with PV-loop data is essentially optimal for classification and can, therefore, be used as a ceiling to infer early stopping for non-invasive data. Given the postulate of the study, we assert that 97% is the ceiling for cardiac-based predictions of mouse strain in this experimental setting.

### Evaluating the Non-invasive Transthoracic Doppler Echocardiogram

For non-invasive TTDE data to classify mouse strain, the analysis setup was similar to the PV-loop data. Pre-processed data were classified along 15 physiological parameters, four metrics for variability and the number of heartbeats per animal. TTDE data exhibits scale variability due to the physical properties the measurement, therefore, data were normalized to the grand mean and standard deviation prior to training. Without normalization, training was inefficient and inaccurate (shown below). Training was conducted over 2000 epochs and the early-stopping procedures were applied post hoc.

### Early Stopping

**Fig. 4:**
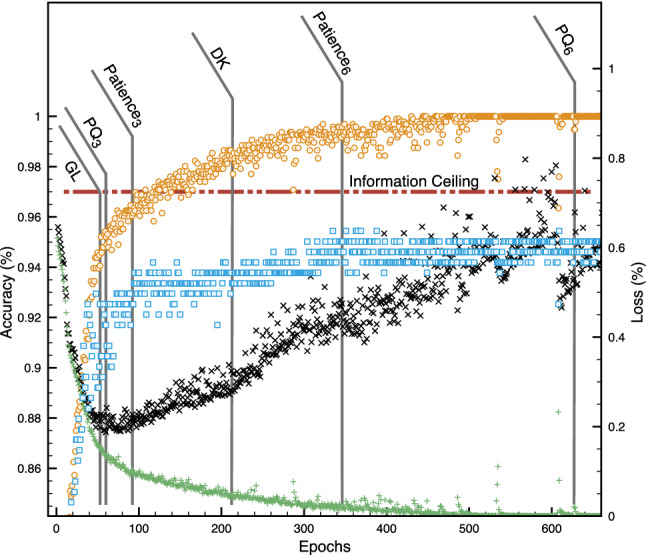
Accuracy (left y-axis) and loss (right y-axis) of the DNN with the training data (tan circles and green plus, respectively) and validation data (blue squares and black x, respectively) by epoch. As expected, the DNN on training data eventually becomes 100% accurate with a steady decrease in loss, due to memorization. Validation accuracy largely levels off, while validation loss reaches a minimum, and then climbs for the remainder of the 2000 epochs (data beyond 660 epochs not shown). Each early stopping rule application (described in the text and Table 2) is indicated at the epoch where the stopping rule was triggered. The best performance is around epoch 100 for generalization error, and the Patience_3_ procedure was the closest to that ideal in this scenario. Training the DNN beyond the invasively determined information ceiling at 97% (horizontal brown dashed line) should be impossible without overfitting by learning training-data-specific features. Assuming zero information loss in the indirect, non-invasive data, our information-ceiling method would trigger stopping at approximately 120 epochs. Image from Bartlett et al. [1]

We applied several early-stopping guidelines based on metrics and heuristics from the literature to assess how each performed in this setting and whether they could be misleading. In addition, we used the empirical ceiling (97%) for additional guidance. The patience parameter is commonly used in the literature with values of 3 or 6 (Patience_3_ and Patience_6_ in Table 2). We also used the Generalization Loss (GL in Table 2) metric which is a function of the loss function value in a given iteration divided by the minimum loss observed in any previous epoch [2]. We chose a value that was 5% of the initial loss. The Progress Quotient is a function of the Generalization Loss smoothed over a strip of *N* previous iterations [2]. We chose *N* to be 3, and 6 (PQ_3_ and PQ_6_ in Table 2), to be comparable to our selected patience values. Lastly we implemented an early-stopping procedure from a non-medical context that modifies the patience parameter dynamically based on the loss from the latest iteration [3]. If the validation loss is smaller than 0.996 of the lowest observed up to that point, then the patience is increased by 0.3 times the current number of iterations. Training stops when patience is less than the current number of iterations (DK in Table 2). Accuracy from the various early-stopping procedures is summarized in Table 2, and the per-epoch accuracy and loss are shown in Fig. 4.

**Table 2:** Summary of DNN training results by stopping rule

	#	Test	Test
		Accuracy %	Accuracy %
	Epochs	(Subset)	(Novel)
GL	54	0.904	0.979
PQ_3_	61	0.909	0.975
PQ_6_	629	0.895	0.950
Patience_3_	93	0.946	0.977
Patience_6_	345	0.925	0.925
DK	212	0.946	0.975

Note that Test Accuracy (subset) refers to hold out data from animals that were in the training data while Test Accuracy (novel) refers to data from hold-out animals that had no data in the training, validation, or test sets

On the unnormalized data, the best validation accuracy was 0.752 across 2000 training epochs. Given the disparity with the normalized data, we did not analyze early-stopping heuristics. This result highlights the critical need for pre-processing to reduce non-biological sources of variation in the biomedical data for this classification task.

#### Prediction from Combined PV-Loop and TTDE Data

Merging the PV-loop and TTDE DNNs into a single network did not improve classification (96.5%) over PV-loop data alone (97%)—which are the same accuracy within the variability of the design—using the same early-stopping rule as employed in the PV-loop only analysis. These results indicate that no additional information useful for the classification task is present in the non-invasive measurement.

### Conceptual Generalization of the Invasive/Non-invasive Dichotomy

**Fig. 5:**
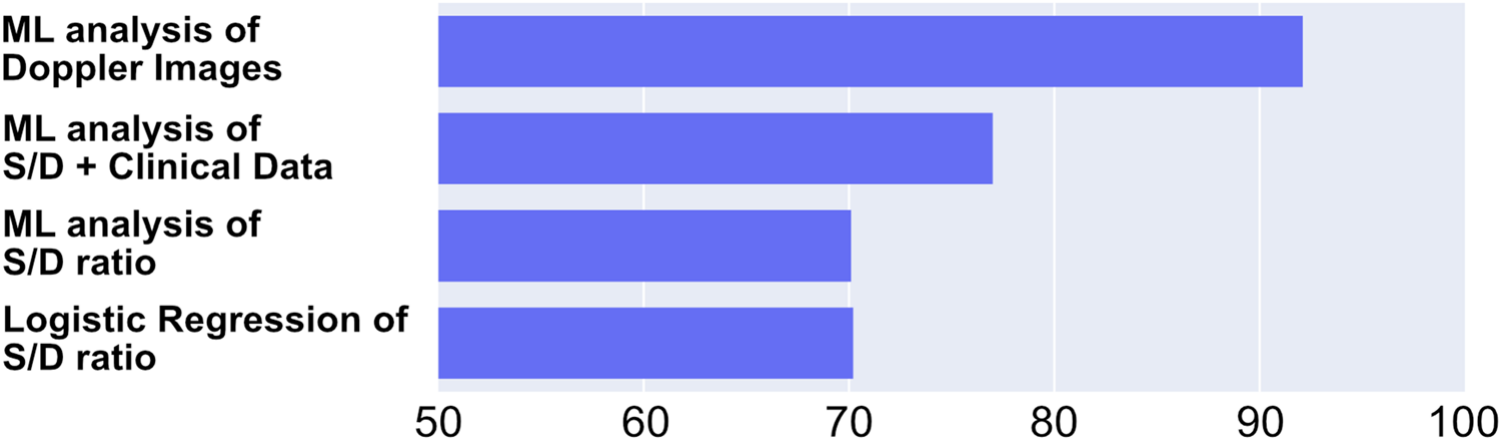
The sonographic images distinguish between IUGR fetuses vs. control using the Xception DNN architecture (ML analysis of Doppler Images), while the systolic/diastolic (S/D) ratio alone, or the S/D data and the clinical data, both have predictive performance that is markedly reduced compared to the image analysis. These data indicate that the Doppler alone contains, and ML can effectively extract, predictive information not previously available in routine clinical work

We applied the same paradigm to a physiology versus clinical data dichotomy based on the same principle that, at least in this case, the physiological measurement is closer to the underlying pathophysiology than the routine clinical data included in this study. To classify IUGR using umbilical artery Doppler flow, we applied a well-tested convolutional neural network architecture (Xception) originally developed by Google. The network was trained on a small subset of 423 Doppler flow images from 169 patients. A validation dataset of 60 images was used to monitor training progress. The final test dataset of 29 images from 29 patients (not included in training or validation data) showed 93.1% accuracy. By way of comparison, the ratio of Doppler flow during systolic versus diastolic phases of the heartbeat alone attained only 70.2% accuracy on the same patients using a logistic model and 70.1% using a (fully connected) DNN. Including both systolic/diastolic ratio along with the clinical data known before birth to train the DNN, accuracy only increased to 77% as shown in Fig. 5.

Addition of the DNN for the clinical data to the image analysis DNN in an ensemble does not increase accuracy. Taken together, these analyses indicate that the Doppler signal alone contains information that could be useful for clinical diagnostics which is not currently available to clinicians, and that ML can effectively extract this information. As it contains no additional information, training the clinical data DNN past the point of the Doppler flow accuracy would have been a clear example of memorization. As such, the UA Doppler flow results represent an early-stopping ceiling.

## Discussion

In this paper, we develop the idea that an objective ceiling for early stopping using noise-prone, “distant” measurements, could be derived from more direct measurements of an underlying process. In this case, we postulated that an invasive measurement should provide as much, or more predictive power as a non-invasive measurement of the same underlying process. We used data from animal experiments that are part of an ongoing project to study early markers for a type of cardiac disease that affects blood flow. Cardiac catheterization to determine pressure–volume loops is an invasive measurement while sonographic cardiac TTDE is not. The latter is important since non-invasive measurements are preferred for diagnostics in humans and machine learning on diagnostics in humans is an important area for biomedical science.

Yet, early stopping for noisy biomedical measurements in real-world applications relies on the same ad hoc procedures as other machine learning applications. Though biomedical datasets are often expensive to obtain and difficult to effectively work with, perhaps in one way biomedical data have an advantage over naturalistic data from, for example, internet traffic-derived information. Biomedical sciences can perform experiments that clearly delineate direct measurements of an underlying biological process from indirect measurements of the same process. Given the precept guiding this work, it is unlikely that non-invasive measurements will outperform invasive measurements based in machine learning applications. Any time accuracy in the non-invasive training dataset exceeds the invasive performance ceiling, we can be sure that modeling is overtraining and an early-stopping rule needs to be chosen to find a stopping point with less generalization error.

Notably, stopping based on our criteria of training until the non-invasive dataset reaches the invasive performance (97%), would result in stopping training in this experiment at approximately 120 epochs, which is just past the point (approximately 100 epochs) when validation loss begins to climb. If one assumes as a heuristic that *some* information loss occurs in the indirect (non-invasive) measurement compared to the direct (invasive) measurement, a ceiling might be specified slightly below that determined from the invasive data, resulting in stopping somewhat earlier. This is near-ideal for this dataset.

Could the objective performance ceiling come from animals and applied to non-invasive human data? While this is tempting as a possible general rule, there are key differences between animals and humans that preclude strong advice. In our setting, we note that the animal models of cardiac function are indeed very similar in important ways to humans but the measurements offer a few distinct differences. First, the size of the mouse heart is much smaller. The ultrasound measurement procedure will have somewhat different noise issues. For example, given the size of the heart, noise is introduced based on the orientation of the ultrasound probe that is much greater than would be seen in humans. Second, the animals are sedated during the sonographic TTDE acquisition, where humans would not be. Third, in human data, it may be possible to improve classification results beyond what is shown here using other clinical variables (such age, sex, and other diagnosed diseases).

We postulate that when multiple approaches are available to evaluate a system, results from a more direct measurement may be used to define an information ceiling for the less direct measurements. In the bio/life sciences, it is common for there to be many different ways to measure a phenomenon, ranging from inexpensive indirect inferential measurements to expensive direct invasive measurements. We suggest that the results of the expensive direct invasive measurements, which are frequently available in the literature, may be used to define informational ceilings for machine learning on the less expensive, indirect measurements. Overall, this study is an example that offers an additional guidance possibility for machine learning researchers working in biomedical research or other similar experimental contexts.

## Conclusions

This study provides an evidence base to develop best practices for early stopping when training deep neural networks. While patience is commonly available in DNN packages, there is a short list of competing metrics that we included in our study. We showed that the early-stopping metrics have great variability in performance. Our proposal was to use exogenous information to know when to stop training. For biological data, it is possible that training is done on more direct data, i.e., data collected the closest to the underlying physiology as possible, can be used to provide an objective ceiling for training. The logic model assumes that more indirect data sources must contain more noise and, therefore, cannot be used to train a DNN that outperforms a superior (more direct) dataset. We showed this principle works well in two biological settings, first, invasive measures versus non-invasive measures and second, a more direct physiology measurement versus a (less direct) clinical judgment. In both cases, early stopping based on the more direct measurement performed well.
